# Biofilm and hemagglutinin formation: a Hallmark for drug resistant uropathogenic *Escherichia coli*

**DOI:** 10.1186/s13104-019-4382-1

**Published:** 2019-06-24

**Authors:** Dawit Gebreegziabiher Hagos, Tadele Araya Mezgebo, Samuel Berhane, Araya Abraha Medhanyie

**Affiliations:** 1 Department of Microbiology and Immunology, College of Health Sciences, Mekelle University, Mekelle, Tigray Ethiopia; 2 Department of Internal Medicine, College of Health Sciences, Mekelle University, Mekelle, Tigray Ethiopia; 3 School of Public Health, College of Health Sciences, Mekelle University, Mekelle, Tigray Ethiopia

**Keywords:** UPEC, Biofilm, Drug resistance, UTI

## Abstract

**Objective:**

Urinary tract infection (UTI) is one of the most frequent disease encounters in pregnant mothers, and the most drug resistant, biofilm and hemagglutinin producer Uropathogenic *Escherichia coli* (UPEC) is the major etiologic agent. Therefore, the aim of this study was to assess the association between the antimicrobial resistance, and biofilm and hemagglutinin production of Uropathogenic *Escherichia coli*.

**Results:**

UTI among the study participants was 27.3%; and UPEC was found the major etiologic agent followed by coagulase negative staphylococcus. Risk factors, previous history of catheterization and previous history of UTI were found significantly associated with UTI, recurrent UTI, drug resistance and biofilm formation. Of the tested antibiotics, nitrofurantoin was the most effective drug for UPEC. Nearly 100% of the biofilm producers were resistant to norfloxacin, cotrimoxazole, and gentamicin.

## Introduction

UTI is one of the major bacterial infections worldwide and about 50% of women experience at least one episode during their lifetime [1–3]. Nearly 25% [4] and 44% [5] of women develop recurrent UTI within 12 months following acute infection, resulting an estimated prevalence of 150 million cases globally per year [4]. Recurrent UTI contributes significantly to UTI associated morbidity and demands huge resources [4, 5]. For example, in the United States of America (USA), more than 7 million people visit health institutes every year and the costs to manage it reaches approximately over $1 billion every year [6]. Moreover, UTI is among the highest antibiotics prescribed diseases in the health institutions around the worldwide [7, 8].

Though there is no enough and well documented data in developing, a few studies indicated that UTI is one of the top causes of morbidity [9–11]. A community based study in Nigeria showed that the prevalence of UTI is about 13%, of which majority of this was due to UPEC [12]. Likewise, in Ethiopia, very limited studies indicated the same trend with other developing countries. A study done at Tikur Anbessa Specialized Teaching Hospital, Addis Ababa, Ethiopia indicated that the overall prevalence of UTI is 23.32%, where *E. coli* was the major isolate [13, 14]. Other studies at Felge Hiwot Referral Hospital, Bahirdar and Hawassa Referral Hospital, also showed that the prevalence of UTI was 30.2% [14] and 48% [15] respectively. Furthermore, the burden of UTI is not only morbidity, mortality and economic impact at individual and nationwide, but it also incurs a huge antibiotic consumption and hence one of the causes for increasing drug resistance to the commonly prescribed drugs [1].

Over 80% [9, 10] of the etiologic agent of UTI is UPEC [16]. Frequently, the patient’s own intestinal flora is the source of infection [6, 9]. In addition to the ability of UPEC to firmly attach to the urinary bladder and kidney, biofilm formation is also considered another pathogenic determinant, which allows UPEC to persist for a long time in the urinary tract and interfere with bacterial elimination [17]. Biofilms can define as structured bacterial communities embedded in a self-produced exopolysaccharide matrix adherent to any abiotic or biological surface [12, 18]. Currently, antimicrobial resistance is one of major health threats and it is even more in developing countries, where no strict drug monitoring program. The problem is very much significant in UTI; as the major cause (UPEC) is one of the most drug resistant pathogens.

The microbes have evolved a number of mechanisms to evade antimicrobial therapy and the most important way for UPEC is the ability to form the biofilm [19]. Biofilm endows bacteria with several advantages, such as the acquisition of antibiotic tolerance, expression of several virulence factors and increased resistance against phagocytosis and other host defense mechanisms [18]. Studies comparing biofilm positive versus biofilm negative UPEC strains showed that, drug resistance was significantly higher in vitro biofilm formers than non-former [3, 18]. Other studies also indicated that more UPEC drug resistance was observed in strains which produce hemagglutinin [9]. Therefore, the objective of the study was to assess if the very drug resistant nature of UPEC is associated with biofilm formation and hemagglutinin production.

## Main text

### Method

This cross-sectional study was conducted to assess the association between antimicrobial resistance and biofilm formation and hemagglutinin production of the most eminent drug resistant Uropathogenic pathogens at Mekelle University, College of Health Sciences, Ayder Comprehensive Specialized hospital, Antenatal Care (ANC) Clinics from December 2017 to August 2018. Using a structured questionnaire-based interview, demographic and clinical data were collected from 323 study participants. Training was given to data collectors on how to collect the midstream urine and data. The midstream urine was collected using wide-mouthed and clean container and transported to Mekelle University, College of Health Sciences, Medical Microbiology laboratory within 1 h of collection and cultured to isolate the etiologic agents. The specimen was cultured on CHROMaga, at 37 °C for 24 h aerobically. The bacterial isolates were further identified by standard biochemical tests [1, 2]. On the UPEC isolates, hemagglutination test was performed by mixing one drop of bacterial suspension with one drop of 3% blood group “O” red blood cells in phosphate-buffered saline (PBS) with and without 3% mannose [4, 6]. Biofilm formation was also tested by Congo red agar (CRA) method. The culture media contain brain heart infusion broth 37 g/L, sucrose 50 g/L, agar10 g/L and Congo red 8 g/L. Plates were inoculated and incubated aerobically for 24 h at 37 °C [5, 6].

On Muller Hinton agar, Kirby-Bauer disc diffusion assay was carried out to determine the antimicrobial susceptibility profiles [3]. Based on the frequency of drug prescription in the study area, the following antibiotics (Oxoid UK) were included: amoxicillin: 30 mg, gentamicin: 10 mg, cefotaxime: 30 mg, nalidixic: acid 30 mg, ciprofloxacin: 5 mg, ofloxacin: 5 mg, norfloxacin: 10 mg, erythromycin: 15 mg, oxacillin: 5 mg, vancomycin: 30 mg, nitrofurantoin: 300 mg and tetracycline: 30 mg. Quality control strains; *S. aureu*s (ATCC 25923) and *E. coli* (ATCC 25922) were used as a quality control for culture and susceptibility testing throughout the study. Data analysis was done by SPSS Ver. 20 and descriptive and multivariate analysis was performed. Those variables which were statistically significant, (*p *< *0.05*) in bivariate logistic regression were moved to multivariate logistic regression.

### Results

A total of 323 pregnant women with symptomatic of UTI were included in this study. Median age of the study subjects were 24 ± 5.4 years old and 297 (91.9%) were urban residents (Table 1). Among the study subjects, 283 (87.6%) were married, and about 176 (54.5%) were house wives. Regarding educational status, about 47 (13.2%) and 55 (17%) of them were illiterate and attended college education (Table 1).

**Table 1:** Socio-demographic characteristics of the study participants in Ayder Comprehensive Specialized Hospital December 2017–August 2018 (n = 323)

Variables	Frequency	Percent (%)
Age (years)		
15–24	134	41.5
25–34	169	52.3
35–44	20	6.2
Residence		
Urban	297	91.9
Rural	26	8.1
Occupation		
House wife	176	54.5
Merchant	54	16.7
Student	12	3.7
Government employee	81	25.1
Educational level		
Illiterate	47	14.6
Only read and write	26	8.1
Primary school	81	25.1
Secondary school	114	35.2
College and above	55	17.0
Marital status		
Single	15	4.6
Married	283	87.6
Divorced	8	2.5
Widowed	7	2.2
Others	6	3.3

Of the total, bacteriuria was detected in 87 (26.9%) of the study participants. In multivariate analysis previous history of UTI (AOR = 7.057, 95% CI (5.269, 18.654), P = 0.002) and previous history of catheterization (AOR = 2.870, 95% CI (1.516, 9.122), P = 0.003) were significantly associated with UTI. Though the number of patients with previous history of catheterization were small 23 (7.1%), it was found significantly associated with UTI (P = 0.03). Variables like marital status, residency, education status, other chronic diseases like diabetic mellitus and previous hospitalization were not showed a significant association with UTI (Table 2).

**Table 2:** Bivariate and multivariate logistic regression analysis of factors associated with UTI among pregnant women attending ANC clinic of ACSH, December 2017–August 2018

Variables	Culture		COR (95% CI)	P-value	AOR (95% CI)	P-value
	SB negative no. (%)	SB positive no. (%)				
Educational level						
Illiterate	28 (59.6)	19 (40.4)	2.07 (0.23, 7.008)	0.078	3.001 (0.702, 16.903)	0.115
Only write & write	11 (53.4)	10 (47.6)	1.711 (0.513, 7.940)	0.234	3.975 (0.574, 22.876)	0.110
Primary school	55 (73.3)	20 (26.7)	1.561 (0.685, 3.987)	0.209	2.421 (0.692, 9.759)	0.170
Secondary school	69 (75.0)	23 (25.0)	1.602 (0.573, 4.112)	0.363	1.989 (0.766, 7.095)	0.152
Higher education	77 (83.7)	15 (16.3)	1			
History of UTI						
Yes	81 (71.1)	33 (28.9)	9.932 (4.904, 20.114)	0.001	8.015 (4.005, 19.908)	0.001*
No	201 (96.2)	8 (3.8)	1			
History of catheterization						
Yes	86 (83.5)	17 (16.5)	6.003 (3.113, 10.976)	0.002	3.270 (1.316, 8.122)	0.012*
No	214 (97.3)	6 (2.7)	1			
History of hospitalization (last 3 months)						
Yes	62 (78.5)	17 (21.5)	4.038 (1.843, 8.633)	0.032	3.102 (0.535, 11.765)	0.348
No	201 (82.4)	43 (17.6)	1			
History of antibiotic use (last 3 months)						
Yes	37 (61.7)	23 (38.3)	1.227 (2.007, 6.406)	0.057	2.102 (0.724, 3.935)	0.834
No	217 (82.5)	46 (17.5)	1			

*SB* Significant bacteriuria, *ACSH* Ayder Comprehensive Specialized Hospital, *COR* crude odds ratio, *AOR* adjusted odds ratio

Five bacterial species were isolated and of these with significant bacteriuria, 94.8% were had a single isolate, while the remaining 5.2% were dually infected. Out of the total isolates, *E. coli* was the major isolated, which is followed by coagulase negative staphylococci (CoNS), *S. aureus*, and *K. pneumonia*. Majority of the isolates were gram negative uropathogens and the resistance rate against Ciprofloxacin, Tetracycline, Trimethoprim-Sulfamethoxazole, Ceftriaxone, Amoxacillin-Clavulinic acid, Norfloxacin, Cotrimoxazole, Cefotaxime, Amikacin, Gentamicin; range from 27.0 to 100%. However, all Gram-negative bacterial isolates were showed relatively low level of resistance to nitrofurantoin (3.8%) and ceftazidime (17%).

The leading isolate *UPEC*, showed high level of resistance to Ampicilin 13 (33.3%) and tetracycline 9 (23.1%). On contrary, 76.5% of the UPEC were relatively sensitive to ceftazidime and norfloxacin each, followed by nitrofurantoin (96.2%). *K. pneumoniae* isolates were 96% resistant to, Gentamicin, Amoxacillin-Clavulinic acid and Trimethoprim-Sulfamethoxazole. Other drugs like Norfloxacin, Amikacin, Nitrofurantoin, Ciprofloxacin, Ceftriaxone, Cefotaxime, Ceftazidime and Ampicillin were resistant to 60.0% of *K. pneumonia* isolates. All the UPEC isolates (n = 39) were tested for biofilm formation and hemagglutins production. Of these, hemagglutin and biofilm formation were detected in 38 (97.4%) and 24 (61.5%) respectively. In this study, 23 (96%) of the biofilm producing UCEC isolates were found Multi-drug Resistant (MDR). More interestingly, all biofilm producing UPEC were found 100% resistant to antibiotic like norfloxacin, cotrimoxazole, and gentamicin. However, nitrofurantoin was found to be 98% effective to the biofilm producing UPEC. On multivate analysis biofilm and hemagglutins producing UPEC isolates were significantly associated with being MDR (*P *= *0.002*).

### Discussion

The prevalence of UTI in our study was 27.3% and this in agreement with a study done in Addis Ababa 23.3% (25), Nigeria 25.3% [20], Cameroon 23.5% [11], Bar-Dar (30.0%) and lower than the other studies in Dire Dawa 14.4 [21, 22], Tanzania 14.6% [23] and Brazil (15.7%) [24]. On the contrary, our finding was higher from studies done in Bahir Dar 9.5% [21] and Addis Ababa 11.6% [13]. The difference might be due to geographical difference and sample size effect. In addition, the health care system provision, especially the antenatal services are improving from time to time and hence, the time differences might be also the possible explanation for disparities between our result and the reports earlier reports from Ethiopia. In our study, the predominant etiologic agent of UTI was UPEC and this is in agreement with almost all studies done around the world [3, 19, 24, 25].

Previous history of UTI and history of catheterization were significantly associated with UTI and that is similar to studies reported from Bahir Dar [26], and Pakistan [27]. The possible justification for this may be, due to empirical treatment and overuse of drugs. However, maternal age, residence, educational level, marital status and occupation are variables which did not show a significant association with UTI and this was in agreement with reports from Bahir Dar [26], Gondar [14], Sudan [28] and Tanzania [23].

As a result of absence of appropriate diagnostic tools for diagnosis and drug susceptibility testing plus extensive use empirical treatment, development of drug resistance is vertically raising [18]. In our case drug resistance is defined as; if an isolate is resistant to two or more different chemical classes, then we considered as MDR isolate. In the present study, 76.7% of the isolates were found MDR and this was higher than a study report from Addis Ababa, Tikur Anbesa Specialized Hospital [25] and lower than the findings from Gondar [14] and Dire Dawa [22] and might be justified by the frequent and inappropriate use of antibiotics.

Adhesionis is a crucial and primary step for UPEC to establish and cause disease along the urinary tract and this is mediated by type 1 fimbriae. Adhesion to the epithelial cells is very important to resist the host immune response and ascend to the upper parts of the urinary tract. In our study, UPEC positive for hemagglutinin formation were significantly associated with MDR (*p *= *0.001*) and with recurrent UTI (*p *= *0.01*); and this was in agreement with a study conducted in India [18]. Different studies report that the ability of UPEC to cause recurrent UTI is because of their ability to form biofilm on the epithelial layer of urinary tract and inanimate plastic materials. In our study, biofilm formation was detected in about 61.7% and this is in agreement with studies conducted in India (22), and lower than another study conducted in India [29]. The relationship between being drug resistant and biofilm formation was showed a statistically significant relationship (*P *= *0.02*), and this was in agreement with the studies conducted in India [18]. Moreover, in the present study, biofilm formation was significantly associated with history of catheterization and history of previous UTI, which implies, biofilm could be one of the main factors for UPEC to develop drug resistance. However, UPEC was found uniquely sensitive to nitrofurantoin and hence it could be the best choice of drug for patient suffering from recurrent UTI and for those who failed to respond to the current treatment regimen.

## Limitations

The present study did not address the different serotypes of *E. coli.*

## Data Availability

All generated data are included in this article.

## Abbreviations

ANC: antenatal Care
CRA: congo red agar
MDR: multi-drug Resistant
PBS: phosphate-Buffered Saline
UTI: urinary tract infection
UPEC: uropathogenic *Escherichia coli*
USA: United States of America
